# Supplementary figures and images for: ANKRD24 organizes TRIOBP to reinforce stereocilia insertion points
Source: J Cell Biol. 2022 Feb 17;221(4):e202109134. doi: 10.1083/jcb.202109134 (PMC8859912; doi:10.1083/jcb.202109134)

Fig 2M

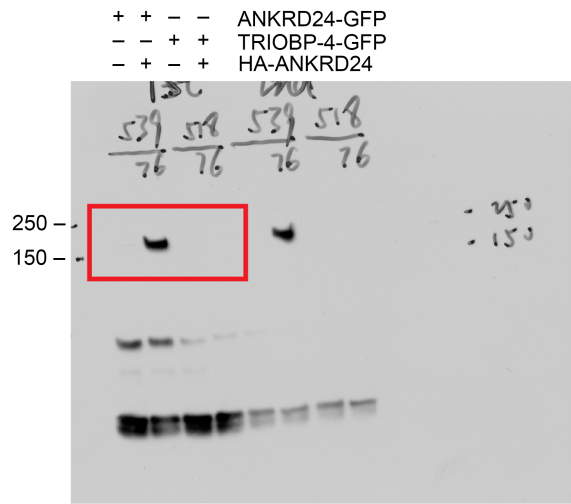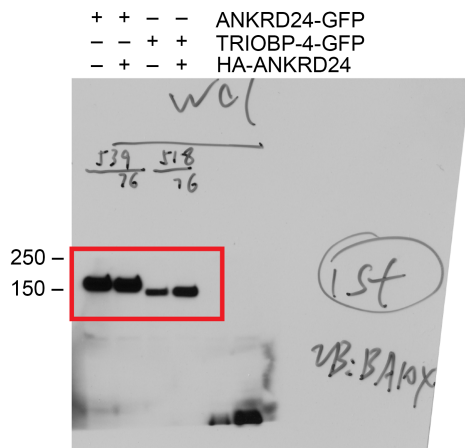

K

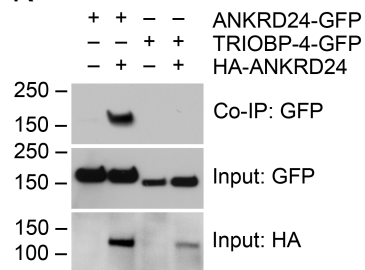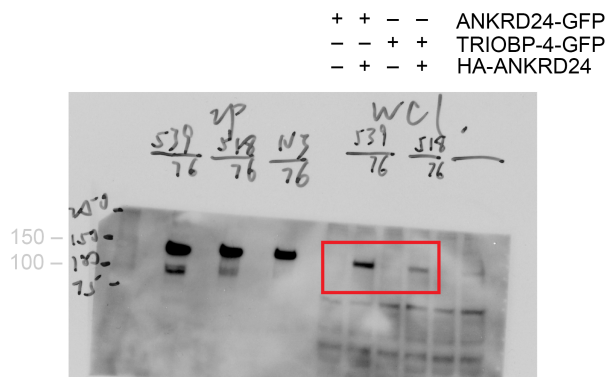

Supplement: SourceData F2 — contains original blots for Fig. 2. [file JCB_202109134_SourceDataF2.pdf]
