# Supplementary material for: ANKRD24 organizes TRIOBP to reinforce stereocilia insertion points
Source: J Cell Biol. 2022 Feb 17;221(4):e202109134. doi: 10.1083/jcb.202109134 (PMC8859912; doi:10.1083/jcb.202109134)
Supplement: SourceData F3 — contains original blots for Fig. 3. [file JCB_202109134_SourceDataF3.pdf]

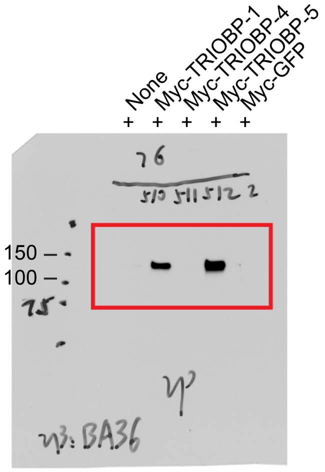

Fig 3B

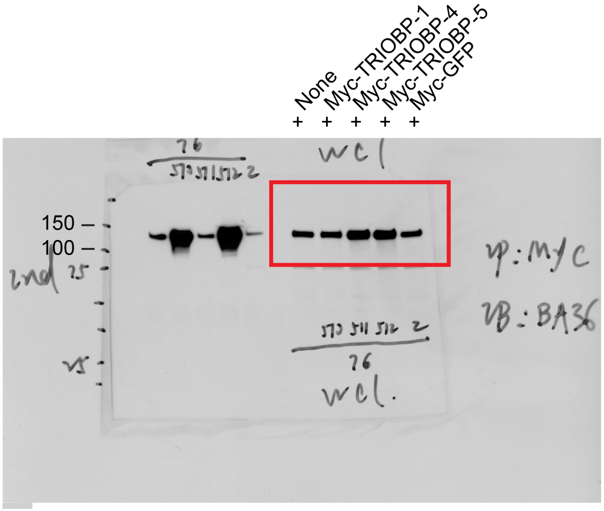

B

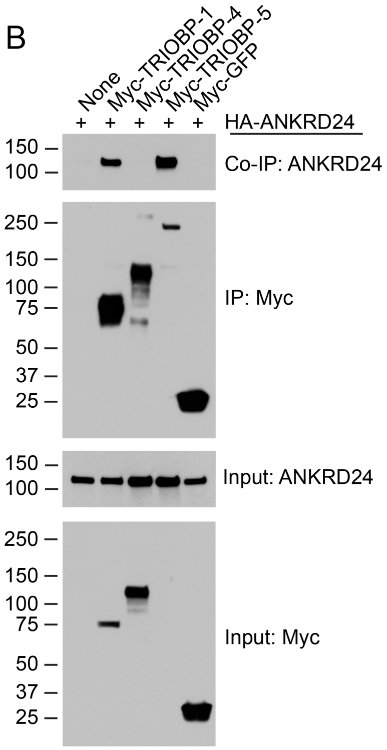

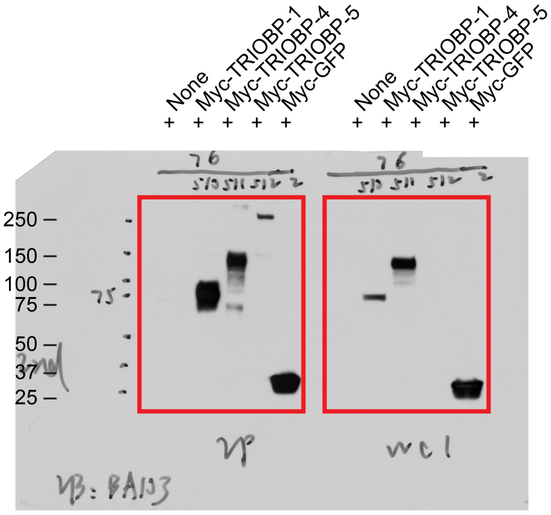

76: HA-Ankrd24
510: Myc-triobp isoform 1
511: Myc-triobp isoform 4
512: Myc-triobp isoform 5
2: Myc-GFP
BA36: anti-HA
BA103: anti-Myc
