# Supplementary material for: ANKRD24 organizes TRIOBP to reinforce stereocilia insertion points
Source: J Cell Biol. 2022 Feb 17;221(4):e202109134. doi: 10.1083/jcb.202109134 (PMC8859912; doi:10.1083/jcb.202109134)
Supplement: SourceData F4 — contains original blots for Fig. 4. [file JCB_202109134_SourceDataF4.pdf]

Fig 4B

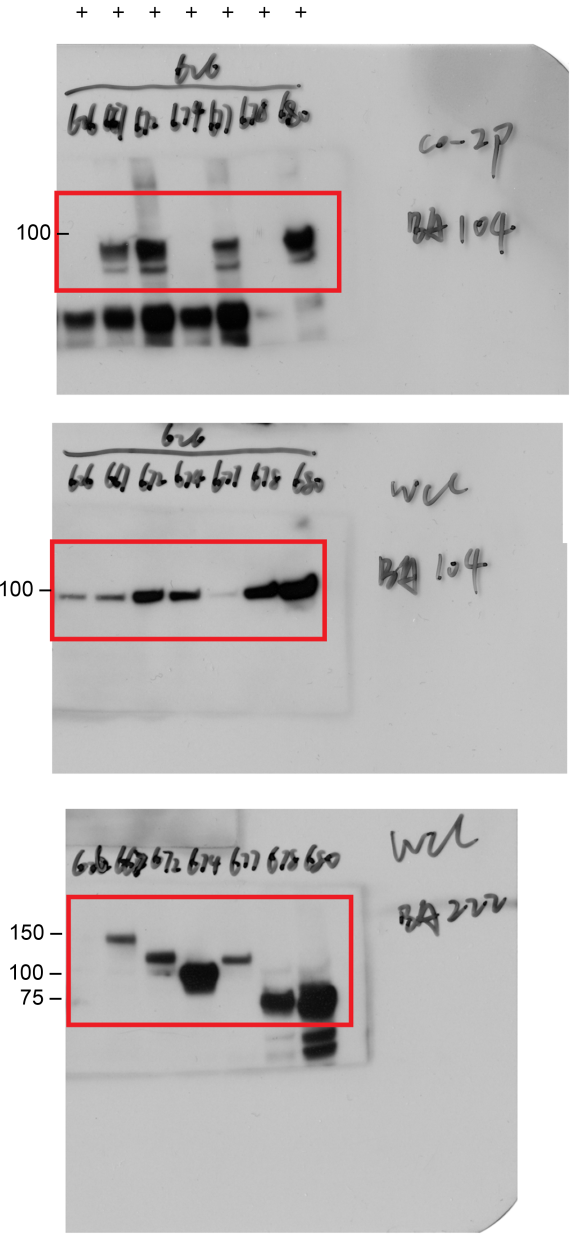

Fig 4C

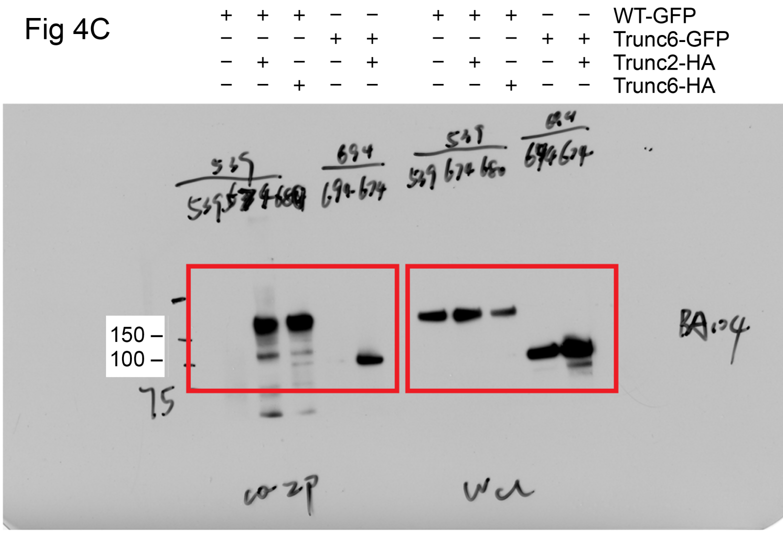

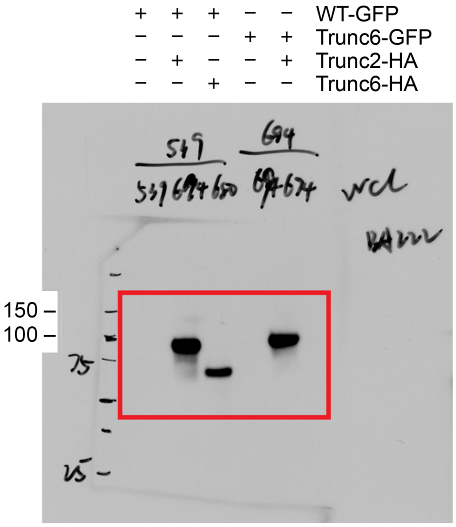

B

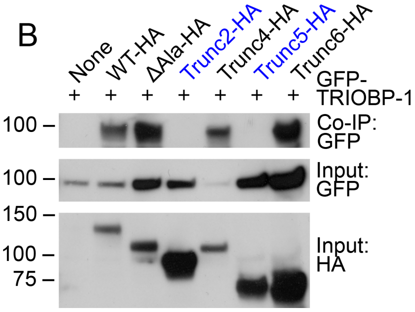

C

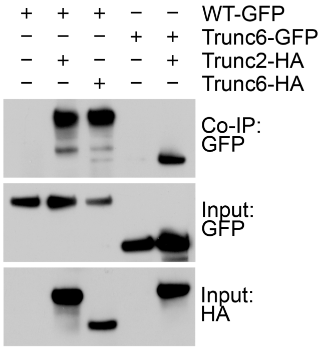

539: ankrd24-GFP
674: Ankrd24 Trunc2 (1-691)-HA
680: Ankrd24 Trunc6 (489-985)-HA
694: Ankrd24 Trunc6 (489-985)-GFP
BA104: anti-GFP
BA222: anti-HA
